# Human retinal endothelial cells express functional interleukin-6 receptor

**DOI:** 10.1186/s12348-023-00341-6

**Published:** 2023-04-25

**Authors:** Lisia Barros Ferreira, Liam M. Ashander, Binoy Appukuttan, Yuefang Ma, Keryn A. Williams, Giles Best, Justine R. Smith

**Affiliations:** Flinders University College of Medicine and Public Health, Flinders Medical Centre, Rm 4E-431, Bedford Park, Adelaide, SA 5042 Australia

**Keywords:** Uveitis, Retina, Endothelial cell, Human, Interleukin-6, Receptor

## Abstract

**Background:**

Interleukin (IL)-6 is an inflammatory cytokine present in the eye during non-infectious uveitis, where it contributes to the progression of inflammation. There are two major IL-6 signaling pathways: classic signaling and trans-signaling. Classic signaling requires cellular expression of the IL-6 receptor (IL-6R), which exists in membrane-bound (mIL-6R) and soluble (sIL-6R) forms. Prevailing dogma is that vascular endothelial cells do not produce IL-6R, relying on trans-signaling during inflammation. However, the literature is inconsistent, including with respect to human retinal endothelial cells.

**Findings:**

We examined IL-6R transcript and protein expression in multiple primary human retinal endothelial cell isolates, and assessed the effect of IL-6 on the transcellular electrical resistance of monolayers. Using reverse transcription-polymerase chain reaction, IL-6R, mIL-6R and sIL-6R transcripts were amplified in 6  primary human retinal endothelial isolates. Flow cytometry on 5 primary human retinal endothelial cell isolates under non-permeabilizing conditions and following permeabilization demonstrated intracellular stores of IL-6R and the presence of mIL-6R. When measured in real-time, transcellular electrical resistance of an expanded human retinal endothelial cell isolate, also shown to express IL-6R, decreased significantly on treatment with recombinant IL-6 in comparison to non-treated cells across 5 independent experiments.

**Conclusions:**

Our findings indicate that human retinal endothelial cells produce IL-6R transcript and functional IL-6R protein. The potential for classic signaling in human retinal endothelial cells has implications for the development of therapeutics targeted against IL-6-mediated pathology in non-infectious uveitis.

## Introduction

Interleukin (IL)-6 is an inflammatory cytokine that is present in the eye during immune-mediated or non-infectious uveitis, where it contributes to the progression of inflammation [1–3]. Retinal oedema is the most frequent cause of vision loss in non-infectious uveitis, commonly localized to the macula but also involving the neural retina more diffusely, and associated with breakdown of the blood-retinal barrier [4, 5]. Biologic drugs that target IL-6 were introduced into clinical practice for non-infectious uveitis approximately 10 years ago, and multiple studies have shown beneficial effects in patients; specific therapeutic effects of the standard blocking agent, tocilizumab – which targets IL-6 signaling – include resolution of macular oedema and retinal vasculitis [6–8].

There are two major IL-6 signaling pathways: classic signaling and trans-signaling [9]. The IL-6 receptor (IL-6R or CD126) exists in membrane-bound and soluble forms, the latter generated largely by proteolysis of the former, but also through alternative splicing of the primary gene transcript. Membrane-bound IL-6R (mIL-6R) has restricted cellular expression, described in a 2022 review in *Annual Review of Immunology* [10] as being present on certain leukocyte subsets, hepatocytes and epithelial cells. Complexes of IL-6 and mIL-6R associate with cell-surface gp130 or CD130, which mediates classic intracellular signal transduction in these cells. However, gp130 is expressed on *all* cells, including retinal endothelial cells [11], and can bind complexes of IL-6 and soluble IL-6 receptor (sIL-6R). The sIL-6R is shed from leukocytes during inflammation to trigger intracellular signaling more generally, referred to as trans-signaling. Classic signaling has been linked to reparative properties of IL-6, while trans-signaling has been associated with inflammatory responses [9].

The prevailing dogma is that vascular endothelial cells do not respond to IL-6 thorough the classic pathway, and instead rely on trans-signaling during inflammation. However, the literature is inconsistent, including with respect to *retinal* endothelial cells, a key component of the inner blood-retinal barrier. We sought to address this debate by examining IL-6R expression in multiple primary human retinal endothelial cell isolates, and by assessing the direct effect of IL-6 on the barrier function of human retinal endothelial cell monolayers.

## Materials and methods

### Interleukin-6 and primary antibodies

Human recombinant IL-6 was sourced from Pepro Tech (Rocky Hill, NJ, catalogue number 200–06). Allophycocyanin (APC)-tagged mouse anti-human IL-6R IgG1κ antibody (catalogue number 352805) and fluorescein isothiocyanate (FITC)-tagged mouse anti-human CD31 IgG1κ antibody (catalogue number 303103) were obtained from BioLegend (San Diego, CA).

### Isolation of human retinal endothelial cells

Human retinal endothelial cell isolates were prepared from 11 paired human cadaveric eyes (3 men and 8 women; mean age at death = 55 years; mean time from death to isolation = 27 h), provided by the Eyebank of South Australia (Adelaide, Australia).

Isolation methods and phenotype of the human retinal endothelial cells have been described previously [12–14]. In brief, retinae were dissected from the posterior eyecup, digested with 0.5 mg/ml collagenase II (Thermo Fisher Scientific-Gibco, Grand Island, NY), and cultured in MDCB-131 medium (Merck-Sigma Aldrich, St. Louis, MO) with 2—10% fetal bovine serum (FBS) (Thermo Fisher Scientific-Gibco or GE Healthcare-Hyclone, Logan, UT) and endothelial growth factors (EGM-2 SingleQuots supplement, omitting FBS, hydrocortisone and gentamicin; Clonetics-Lonza, Walkersville, MD) at 37 °C and 5% CO_2_ in air. Cell purification was performed using Dynabeads M-450 Epoxy magnetic beads (Thermo Fisher Scientific-DYNAL, Oslo, Norway) coated with anti-human CD31 antibody (BD Pharmingen, San Jose, CA). The human retinal endothelial cells used for some of this work were expanded by transduction with the LXSN16E6E7 mouse retroviral construct (gifted by Denise A. Galloway, Fred Hutchinson Cancer Institute, Seattle, WA). Importantly, expanded cell isolates retain an endothelial cell phenotype [14].

### RNA extraction and reverse transcription

Cell isolates were plated for confluence in 12-well dishes (growth area = 380 mm^2^; Costar, Corning, NY), held in fresh modified MCDB-131 medium with 10% FBS at 37 °C and 5% CO_2_ in air for 24 h, and subsequently lysed with Lysis Solution supplemented with beta-mercaptoethanol (Merck-Sigma Aldrich) and stored at -80 °C. RNA was extracted using the GenElute Mammalian Total RNA Miniprep Kit (Merck-Sigma Aldrich) or TRIzol Reagent (Thermo Fisher Scientific-Ambion, Carlsbad, CA), following the manufacturer’s guidelines.

Reverse transcription was performed using the iScript Reverse Transcription Supermix for RT-qPCR (Bio-Rad, Hercules, CA), with 100 or 200 ng of RNA input yielding 20 μL of cDNA per reaction. Duplicate reactions were pooled for each sample, and diluted up to 1 in 10 with nuclease-free water for the polymerase chain reaction (PCR).

### Standard polymerase chain reaction

Standard PCR was carried out on a T100 Touch Thermocycler (Bio-Rad Laboratories). In addition to nuclease-free water and 1X PCR buffer (Qiagen, Hilden, Germany), each PCR reaction contained: 0.4 mM dNTP mix, 2.0 mM MgCl_2_, 0.8 μM each of forward and reverse primers (Sigma-Genosys, The Woodlands, TX), 0.625 U of Taq DNA polymerase (Qiagen), and up to 1.5 ng of cDNA template. For IL-6R and mIL-6R, a touchdown protocol consisted of: pre-cycling at 95 °C for 5 min; 10 cycles of denaturation at 95 °C for 30 s, annealing at 66 °C for 30 s (decreasing by 1 °C each cycle), and extension at 72 °C for 90 s; followed by 30 cycles of denaturation at 95 °C for 30 s, annealing at 56 °C for 30 s, and extension at 72 °C for 90 s; ending with a 5-min post-extension hold at 72 °C. For sIL-6R, the protocol consisted of: pre-cycling at 95 °C for 5 min; 40 cycles of denaturation at 95 °C for 30 s, annealing at 62 °C for 20 s, and extension for 60 s at 72 °C; and a 5-min post-extension hold at 72 °C. Product sizes were verified by electrophoresis in 2% agarose gel, and product identities were confirmed by sequencing. Primer sequences and the expected molecular weight of the products are shown in Table 1.

**Table 1:** Primer pairs and product sizes for gene transcripts

**Transcript [Reference] **	**Primer pair**		**Product size (bp)**
IL-6R (mIL-6R + sIL-6R) [15]	Forward	5′-GAGGGAGACAGCTCTTTCTAC-3’	240
	Reverse	5′-CCGTTCAGCCCGATATCTGAG-3’	
mIL-6R [16]	Forward	5′-CTCCTCTGCATTGCCATTGT-3′	202
	Reverse	5′-TGTGGCTCGAGGTATTGTCA-3’	
sIL-6R [16]	Forward	5′-CGACAAGCCTCCCAGGTTCA-3′	195
	Reverse	5′-CGGTTGTGGCTCGAGGTATT-3′	
PPIA [17]	Forward	5’-GAGCACTGGAGAGAAAGGATTT-3’	355
	Reverse	5’-GGTGATCTTCTTGCTGGTCTT-3’	
RPLP0 [17]	Forward	5’-GCAGCATCTACAACCCTGAA-3’	235
	Reverse	5’-GCAGATGGATCAGCCAAGAA-3’	

### Quantitative real-time polymerase chain reaction

Quantitative real-time (q)PCR was performed on a CFX Connect Real-Time PCR Detection System (Bio-Rad Laboratories). As well as nuclease-free water, the qPCR reaction contained: 0.375 µM each of forward and reverse primers, 4 µl of SsoAdvanced Universal SYBR Green Supermix (Bio-Rad Laboratories) and up to 20 ng of cDNA template. The protocol included: pre-cycling for 5 min at 95 °C; 40 cycles of denaturation at 95 °C for 30 s, annealing at 60 °C for 30 s (or at 62 °C for 20 s for sIL-6R), and extension at 72 °C for 30 s; and 1 s hold at 75 °C prior to fluorescence reading. Each primer set generated a single melt peak between 70 °C and 95 °C. Relative normalized expression against the geometric mean expression values of peptidylprolyl isomerase A (PPIA) and ribosomal protein lateral stalk subunit P0 (RPLP0) was calculated in CFX Manager software v3.1 (Bio-Rad Laboratories) using the Pfaffl method [18]. Gene-stability measure and coefficient of variation were required to be less than 0.5 and 0.25, respectively, for both reference genes.

### Analysis of interleukin-6 receptor expression by flow cytometry

Cell isolates were plated for confluence in 12-well dishes, held in fresh modified MCDB-131 medium with 10% FBS at 37 °C and 5% CO_2_ in air for 24 h, and subsequently harvested using 0.05% trypsin for 5 min. The cells were transferred to phosphate buffered saline (PBS) containing 1% FBS (PBS-FBS), centrifuged for 5 min at 280 × *g*, and washed twice with fresh PBS-FBS, with or without 0.05% Triton-X as a permeabilization agent. Cells were stained according to the manufacturer’s recommendations, with anti-human IL-6R and CD31 antibodies, conjugated to FITC and APC fluorochromes, respectively. All samples were incubated on ice in the dark for 30 min. The cells were then washed twice with PBS-FBS and fixed with 4% w/v paraformaldehyde for at least 5 min. Data were acquired on a CytoFLEX S flow cytometer (Beckman Coulter, Brea, CA), and analyzed using FlowJo Software v10.7.1 (BD Biosciences, Franklin Lakes, NJ).

### Measurement of transcellular electrical resistance

Transcellular electrical resistance was measured in real-time on the hour using an RTCA iCELLigence instrument (ACEA Biosciences-Agilent, San Diego, CA) and expressed as ‘cell index’, a unitless measure calculated by comparing the resistance of seeded and medium-only wells. Cells were plated for confluence in multi-well E-plates (growth area = 64 mm^2^) and rested for 30 min at 37 °C and in 5% CO_2_ in air, before being placed in the RTCA iCELLigence instrument. After a 24-h incubation, the E-plates were removed from the instrument. Half the medium volume was replaced, and IL-6 was added to treated wells to a final concentration of 20 ng/ml. The E-plates were returned to the instrument, and transcellular electrical resistance was measured for up to 72 h.

### Statistical testing

Statistical analysis was performed in GraphPad Prism v6.04 (La Jolla, CA), with a significant difference between conditions defined by a *p*-value of less than 0.05.

### Human research ethics

Use of human cadaver donor eyes for this research was approved by the Southern Adelaide Clinical Human Research Ethics Committee (protocol number: 175.13).

## Results

**Fig. 1:**
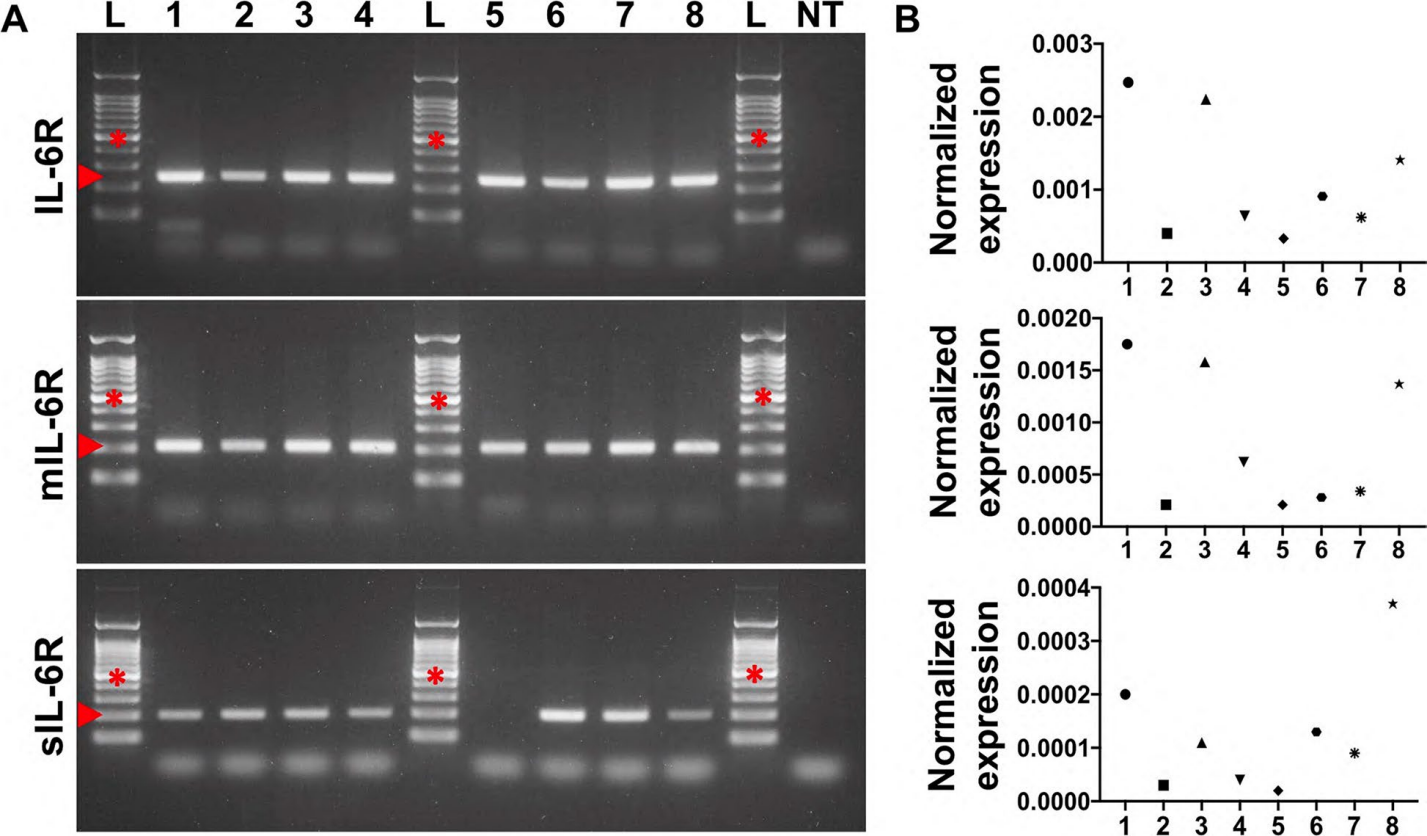
IL-6R transcript expression in human retinal endothelial cells. **A** Images showing IL-6R amplicons run on 2% agarose gel. L: DNA ladder (500 base pairs indicated by red cross); 1–7: primary retinal endothelial cell isolates from individual donors; 8: expanded retinal endothelial cell isolate; NT: no cDNA template control. Expected product sizes (indicated by red arrows): IL-6R: 240 bp; mIL-6R: 202 bp; sIL-6R: 195 bp. **B** Graphs showing relative normalized expression of corresponding IL-6R transcripts in the same cell isolates showed in (**A**). Reference genes were RPLP0 and PPIA

Expression of IL-6R transcript was investigated in human retinal endothelial isolates from 7 donors, including the expanded cell isolate, by standard PCR using primers that detected total IL-6R (forward and reverse primers aligning to exons 4 and 5, respectively), mIL-6R alone (forward primer aligning to exon 9, encoding the transmembrane domain, and reverse primer aligning to exon 10) and sIL-6R alone (forward primer aligning to the splice junction between exons 8 and 10, and the reserve primer aligning to exon 10) (Fig. 1A). An IL-6R product was amplified in all 8 cell isolates. The mIL-6R amplicon was also consistently detected, while the sIL-6R amplicon was detected in 6 of 7 primary isolates, plus the expanded isolate. Relative transcript expression was studied by qPCR using the same primers (Fig. 1B). The IL-6R, mIL-6R and sIL-6R transcripts were amplified for all cell isolates, albeit at varying levels across the samples. Together, these findings confirm that human retinal endothelial cells express transcripts that encode mIL-6R and sIL-6R.

**Fig. 2:**
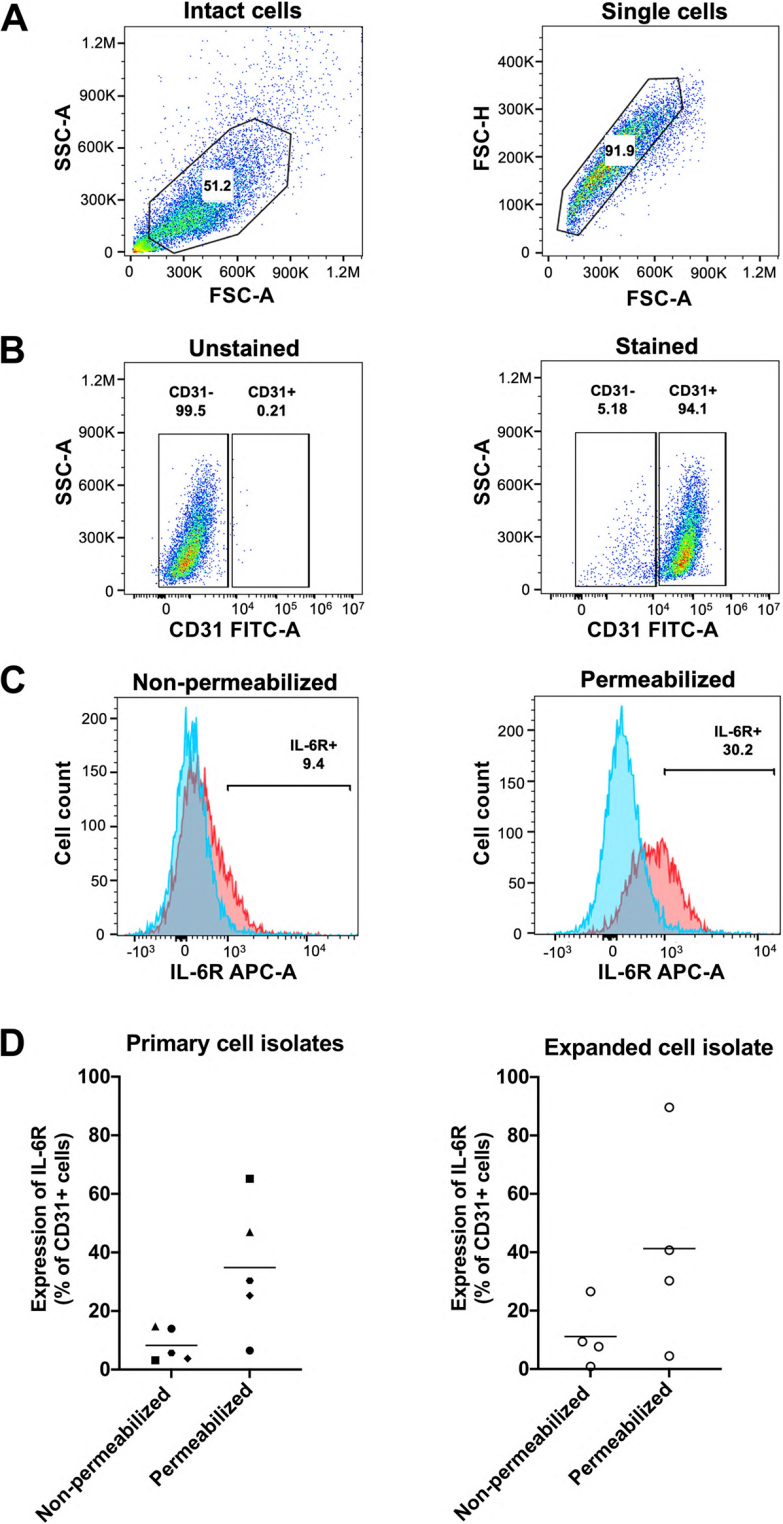
IL-6R protein expression in human retinal endothelial cells. **A-C** Representative flow cytometry plots from one cell isolate: (**A**) Debris and doublets were excluded based on forward scatter (FSC) and side scatter (SSC) properties. **B** CD31-positive were gated in each sample relative to unstained controls. **C** IL-6R expression was assessed in non-permeabilized and permeabilized CD31-positive cells. Blue and red traces indicate the fluorescence in unstained and IL-6R antibody-stained of CD31-positive cells, respectively. **D** Histograms showing percentage of CD31-positive cells expressing IL-6R for individual primary cell isolates (*n* = 5 donors) and the expanded cell isolate (*n* = 4 experiments). Bars indicate mean. Non-permeabilized and permeabilized groups were compared by donor-paired (primary cell isolates) and experiment-paired (expanded cell isolate) 2-tailed Student’s t-test: *p* > 0.05

Presence of IL-6R protein in human retinal endothelial cells was assessed by flow cytometry on 6 cell isolates, including the expanded cell isolate, using non-permeabilized and permeabilized conditions to detect mIL-6R and all IL-6R, respectively (Fig. 2). We selected flow cytometry over Western blot for this work due to the relative sensitivity of the former, and because detection of mIL-6R by the latter would require subcellular fractionation, for which numbers of primary human cells were insufficient. Data were expressed relative to unstained controls. In the primary cell isolates, the mean percentage of positive cells was 8.3% under non-permeabilizing conditions and 34.8% following permeabilization. For the expanded cell isolate, the mean percentage of positive cells was 11.1% under non-permeabilized conditions and 41.3% following permeabilization. These results indicate that human retinal endothelial cells contain intracellular stores of IL-6R and display mIL-6R in the plasma membrane, albeit at relatively low levels across the total cell population.

**Fig. 3:**
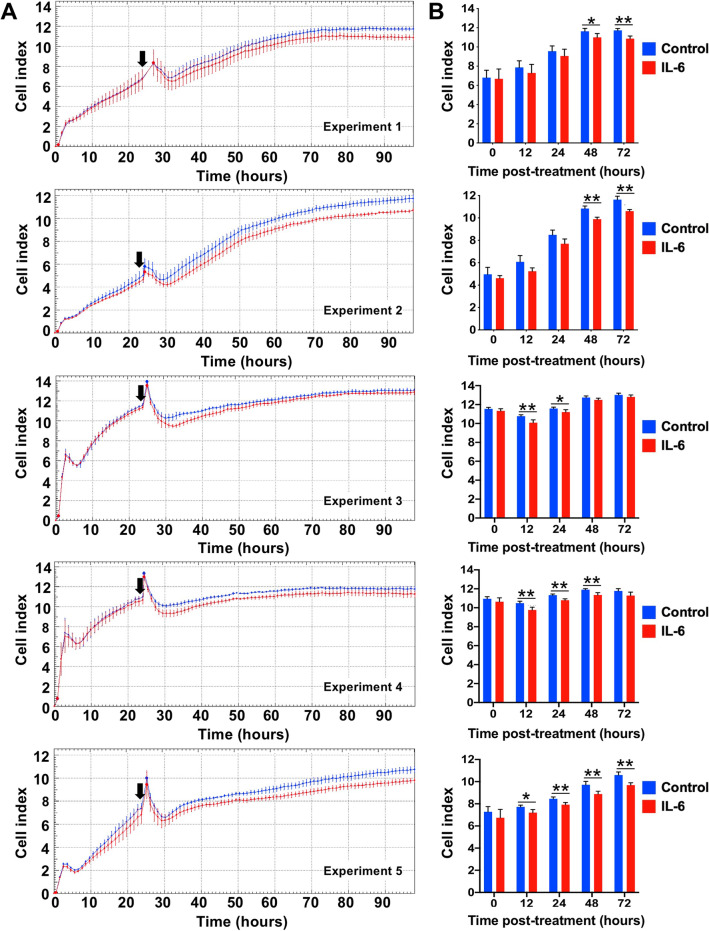
Effect of IL-6 on permeability of human retinal endothelial cell monolayers. Results were generated in 5 independent experiments using the expanded cell isolate. **A** Plots of transcellular electrical resistance across IL-6-treated (red) versus untreated control (blue) cell monolayers, measured as cell index each hour. Arrowheads mark time of IL-6 treatment. Dots represent mean, with error bars indicating standard deviation. *n* = 3–4 monolayers per condition. **B** Graphs showing cell index at specified time intervals following IL-6 treatment for corresponding experiments. Bars represent mean, with error bars showing standard deviation. *n* = 3–4 monolayers per condition. Groups were compared by 2-tailed Student’s t-test: * = *p* < 0.05; ** = *p* ≤ 0.01

To assess the potential function of IL-6R protein in human retinal endothelial cells, the effect of IL-6 on transcellular electrical resistance was evaluated (Fig. 3). These assays required substantial numbers of cells, which necessitated the use of the expanded cell isolate. Across 5 independent experiments, human retinal endothelial cells treated with IL-6 showed a consistent decrease in cell index over time in comparison to non-treated cells, reaching statistical significance across a period of 24 h or more during the course of the 72-h exposure (mean reduction = 7.3%). These observations are consistent with a functional role for IL-6R in human retinal endothelial cells.

## Discussion

Our observations on multiple human retinal endothelial cell isolates indicate that these cells produce mIL-6R and sIL-R transcripts, and functional IL-6R protein.

Current dictum is that vascular endothelial cells do not express the IL-6R, and thus do not activate classic signaling and respond only to IL-6 when also provided with sIL-6R [10]. However, a small number of studies published over the past 30 years suggest otherwise. In 1992, Maruo et al. [19] showed that IL-6 increased the permeability of bovine carotid artery endothelial cells monolayers, and in 2002 Desai et al. [20] showed the same effect in human umbilical vein endothelial cells. Subsequently, Rochfort et al. [21, 22] showed direct effects of IL-6 on expression of junctional complex molecules in human brain endothelial cells. Recently, Zegeye et al. [23] identified the IL-6R on human umbilical vein endothelial cells by flow cytometry, a finding which Montgomery et al. [24] replicated while demonstrating the presence of the IL-6R in coronary arteries of explanted heart transplants. The latter group also presented molecular evidence of classic signaling in human umbilical vein and dermal microvascular endothelial cells.

While much of the ophthalmology literature has supported the dictum, some published work has provided evidence that human retinal endothelial cells might produce IL-6R. On the one hand, Coughlin et al. [25] and Valle et al. [26] could not detect IL-6R expression by human retinal endothelial cells in different protein immunoassays. Furthermore, Da Cunha et al. [27] reported that application of IL-6 to human retinal endothelial cells did not alter transcellular electrical resistance using a biosensor method similar to ours, while Valle et al. [26] needed to add exogenous sIL-6R along with IL-6 to elicit molecular responses from the cells. However in contrast, Yun et al. [28] showed clear molecular responses after direct application of IL-6 to human retinal endothelial cells, and Ye et al. [16] reported the presence of mIL-6R transcript in human retinal endothelial cells, although protein expression was not investigated. Interestingly, Mesquida et al. [29] detected sIL-6R in human retinal endothelial cell culture supernatant, but not to levels that allowed a response to IL-6, and they also could not detect mIL-6R by flow cytometry.

Methodological differences may contribute to variations in findings across groups that work in the area of IL-6 signaling in vascular endothelium. Most research on human retinal endothelial cells has been conducted with single samples, sourced commercially [11]. Our results show that IL-6R is present at variable levels across multiple primary human retinal endothelial cell isolates, and in agreement with Montgomery [24], that it is present at a relatively low level. Interestingly, Zegeye et al. [23] showed expression of IL-6R was down-regulated in human umbilical vein endothelial cells by tumor necrosis factor-α and lipopolysaccharide, an observation that we have also made in human retinal endothelial cells (data not shown). Thus, the activation status of human retinal endothelial cells likely impacts the possibility of demonstrating IL-6R expression.

Our findings could be extended in a multitude of different studies that investigate the molecular consequences of IL-6 signaling. Exploring the outcomes of classic signaling versus trans-signaling in human retinal endothelial cells is important: therapeutics are being developed to differentially target these pathways [30], and outcomes for the retinal endothelium and non-infectious uveitis might be unexpected if both pathways are not taken into consideration. In addition, comparisons between human retinal endothelial cells generated as primary isolates in research laboratories, versus commercially available preparations, may provide valuable information about how these different cells recapitulate IL-6 signaling pathways. Such comparisons may also reconcile the apparently conflicting observations across published studies and aid in the planning of future work.

## Data Availability

All data generated or analysed during this study are included in this published article.

## Abbreviations

IL-6: Interleukin-6
mIL-6R: Membrane-bound IL-6R
sIL-6R: Soluble IL-6 receptor
APC: Allophycocyanin
FITC: Fluorescein isothiocyanate
FBS: Fetal bovine serum
PCR: Polymerase chain reaction
qPCR: Quantitative real-time PCR
PPIA: Peptidylprolyl isomerase A
RPLP0: Ribosomal protein lateral stalk subunit P0
PBS: Phosphate buffered saline
